# The effect of online mindfulness training on connectedness to oneself, to others and to nature in students

**DOI:** 10.1111/aphw.70137

**Published:** 2026-03-10

**Authors:** Petra Jansen, Sabine Hoja, Lea Cassar, Tim‐Michael Neu

**Affiliations:** ^1^ Faculty of Human Sciences University of Regensburg Regensburg Germany; ^2^ Faculty of Economics University of Regensburg Regensburg Germany

**Keywords:** flourishing, inner transformative qualities, mental health, stress, students

## Abstract

These days, students experience a significant amount of stress at university. According to the planetary health concept, internal transformative qualities, such as connectedness, may be essential for enhancing individual well‐being and promoting sustainable behaviour.

This randomised controlled study investigated whether online mindfulness training can enhance well‐being and connectedness. One hundred seventy‐three students completed measurements of stress reduction, flourishing, state mindfulness, self‐compassion, prosociality and connectedness to nature before and after the 8‐week training or the waiting time. The analyses of variances showed a significant improvement in mindfulness (*F*[1, 170] = 5.116, *p* < .05, partial *η* ^2^ = .029), connectedness to nature (*F*[1, 171] = 6.838, *p* < .05, partial *η* ^2^ = .038) and self‐compassion (*F*[1, 171] = 11.019, *p* < .01, partial *η* ^2^ = .061) in the mindfulness group compared to the control group but not in the other dependent measurements. Furthermore, the improvement in self‐compassion and mindfulness from the pretest to the posttest predicted 35.7% and 33.9%, respectively, of the reduction in perceived stress and improvement in flourishing.

Since the mindfulness training only improved some aspects of transformative qualities, it is necessary to investigate the moderators of the relation between mindfulness, internal transformative qualities and sustainable behaviour and attitudes in greater depth.

## INTRODUCTION

Students experience a great deal of stress during their time at the university. While stress is not necessarily harmful, as it can enhance academic motivation (Rahe & Jansen, 2022), it often leads to negative symptoms (Gardani et al., 2022). Additionally, a study conducted in Germany identified students as a high‐risk group for experiencing long‐term psychosocial consequences of the coronavirus pandemic (Werner et al., 2021). Therefore, fostering strategies to reduce stress in the university context appears essential.

One promising approach to stress reduction is mindfulness training. Mindfulness can be understood both as a trait ‐ being nonjudgmentally aware of the present moment (Kabat‐Zinn, 2003) and as a state (Goleman & Davidson, 2017), which can be trained in meditation or, for example, in the mindfulness‐based stress reduction programme (Kabat‐Zinn, 2003). Mindfulness‐based interventions for university students have demonstrated benefits, including reductions in stress, anxiety symptoms, depression and worrying, and an enhancement in well‐being (Dawson et al., 2020). However, evidence suggests that mindfulness‐based stress reduction training may not significantly improve sleep quality, social functioning or subjective well‐being (Pan et al., 2024). This contrasts with another study, in which a specific mindfulness training programme, called Koru, incorporating elements of mindfulness, meditation and mind–body exercises, improved sleep quality in students (Greeson et al., 2014).

Besides this, mindfulness training is also related to connectedness: a brief, 15‐min mindfulness intervention could enhance social and nature connectedness in undergraduate students (Aspy & Proeve, 2017). Connectedness is an internal transformative quality that can influence our values and attitudes towards ourselves and the world (Wamsler et al., 2021). Connectedness is a multidimensional core concept, including connectedness to nature, other human beings (and with the aspects of, for example, empathy and prosociality), the self (which can be measured with the concept of self‐compassion) or something higher (Bucher, 2022). This is partly in line with an assumption of Wamsler et al. (2021), who stated that the concept of connectedness as one part of transformative qualities includes the elements of compassion, empathy, kindness and generosity, which are related to prosociality and connectedness to nature (von Wirth & Jansen, 2025). In this study, we focus on the connectedness to the self (self‐compassion), other human beings (prosociality) and nature.

Besides the relation between mindfulness and connectedness, connectedness is also related to eudemonic well‐being (Pritchard et al., 2020) and flourishing (Rahe & Jansen, 2023). Flourishing can be understood as a broader and more comprehensive concept of well‐being (VanderWeele, 2017). Flourishing encompasses both eudaimonic well‐being—defined as ‘functioning well’—and hedonic well‐being—described as ‘feeling good’ (Schotanus‐Dijkstra et al., 2016). It is often measured through questionnaires that assess factors such as relationship success, self‐esteem and optimism (Diener et al., 2010). Chen et al. (2022) examined the temporal associations between various flourishing domains, including emotional and physical health, meaning and purpose, character strengths, social connectedness and financial security. Each domain contributes independently to an overall flourishing score, with the strongest associations of meaning and purpose, social connectedness and financial security. The importance of social connectedness and a sense of meaning as key components of flourishing is further supported by findings from Rahe and Jansen (2023), who linked flourishing with self‐love and prosociality, a behaviour that benefits others (Eisenberg et al., 2016; Martela & Ryan, 2016)—two vital aspects of connectedness.

Nevertheless, connectedness is not only related to individual well‐being or flourishing but also to actions toward a more sustainable future, or, in other words, to global well‐being. Individuals with higher human‐nature connectedness exhibit more pro‐nature behaviours (Barragan‐Jason et al., 2022), and prosociality and connectedness to nature are positively related to sustainable attitudes and behaviours, and specific aspects of trait mindfulness are linked to the connectedness factors (Jansen, Rahe, & Wolff, 2024). Both studies employ a correlational design, which does not permit causal conclusions. However, fostering sustainable behaviour is not easy because it is related to several factors like values, personal and social norms, one's responsibility, awareness of consequences, attitudes, intentions, perceived behaviour control and habits as described in the comprehensive model of determinants of individual environmentally relevant behaviour (Klöckner, 2013). This model is expanded by including a relational path, emphasising connectedness with nature, empathy and compassion (Thiermann & Sheate, 2020).

One way to foster sustainable behaviour (Fischer et al., 2017) and psychological well‐being (Tang et al., 2019) might be to train inner development or more specific inner transformative qualities, like connectedness. Inner development was neglected for a long time (Ives et al., 2020), but it has recently garnered increased attention (Ives et al., 2023). It aligns with the recently published assessment of the Intergovernmental Science‐Policy Platform on Biodiversity and Ecosystem Services (O'Brien et al., 2024). Inner development, such as compassion, can be trained through mindfulness training (Trautwein et al., 2020). However, it is discussed whether mindfulness training can also enhance sustainable behaviour. The results differ depending on the methods applied:

Geiger et al. (2020) demonstrated that in a sample of university students and employees, a sustainability‐adapted mindfulness‐based intervention did not enhance sustainable consumption behaviour or related attitudes, despite enhancing mindfulness experience. Another study showed that mindfulness and stress reduction training can foster a positive explicit attitude towards vegetarian food as one aspect of sustainable behaviour (Winkelmair & Jansen, 2023) but could not be replicated (Winkelmair & Jansen, 2024). In another experimental study, Riordan et al. (2022) found that participants of an 8‐week MBSR course did not change their eco‐friendly attitudes and behaviour compared to an active control group. It was also shown that mindfulness training can worsen environmentally friendly behaviour (Le Houcq Corbi et al., 2024).

Although the studies on the influence of mindfulness training on sustainable behaviour are not particularly promising, Stenfors et al. (2025) present a conceptual model of the relationships between meditation practice and transformative qualities, including nature connection, mental well‐being and pro‐environmental behaviour. In this model, meditation practice can be directly related to pro‐environmental attitudes and behaviour, or through the mediators of transformative qualities (compassion towards others, compassion towards the self, mindfulness and connectedness to nature), or mental well‐being (mental health continuum and perceived stress). The transformative qualities can also enhance mental well‐being. Stenfors et al. (2025) investigated this theoretical model in a cross‐sectional study involving 185 highly educated decision‐makers in leading positions, working in various sustainability‐related fields across different political institutions. In general, the duration of meditation practice, compassion towards others and connectedness to nature were associated with more pro‐environmental attitudes and behaviours. However, this was not an intervention but a correlational study.

**FIGURE 1:**
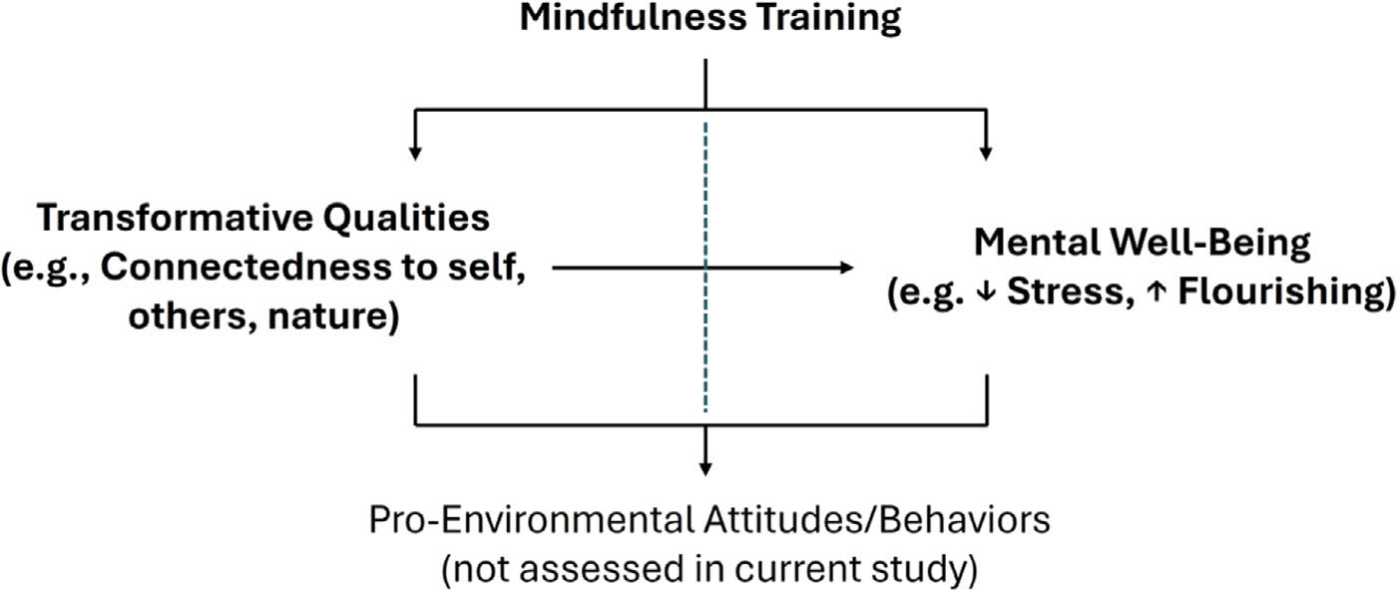
Model of the relationship between mindfulness training, inner transformative qualities and sustainable behaviour. The model (partly in line with Stenfors et al., 2025) shows that mindfulness training can enhance transformative qualities and mental well‐being directly and pro‐environmental attitudes and behaviour directly and indirectly.

The evidence that mindfulness training enhances pro‐environmental attitudes and behaviour is theoretically sound but empirically rare. For this reason, we focus this study on the first part of Stenfors et al.'s (2025) model, whether mindfulness training can foster inner transformative qualities and individual well‐being (see Figure 1).

For the inner transformative qualities, we have chosen the aspect of connectedness, which refers to connectedness to oneself, others and nature. Connectedness was selected because it has been demonstrated in correlational designs that aspects of connectedness are related to sustainable attitudes and behaviours (Jansen, Hoja, & Rahe, 2024a, 2024b). The concept of well‐being is very comprehensive. Pan et al. (2024) refer to subjective well‐being, while Pritchard et al. (2020) employ eudaimonic well‐being in addition to hedonic well‐being or mental well‐being (Stenfors et al., 2025). Stenfors et al. (2025) investigate mental well‐being using the mental health continuum and the Perceived Stress Scale. To also focus on the positive side of well‐being, we examined besides the perceived stress also flourishing. We summarised it under the term ‘individual well‐being’ as a contrast to ‘global well‐being’. Flourishing was chosen because it is a more comprehensive concept of well‐being (VanderWeele, 2017) and encompasses, among other things, happiness, life satisfaction, mental and physical health, meaning and purpose.

The following hypotheses were investigated:

1. An online mindfulness training enhances state mindfulness in students compared to a nonactive control group.
2. An online mindfulness training enhances three aspects of connectedness including self‐compassion, prosocial behaviour and connection to nature in students compared to a nonactive control group.
3. An online mindfulness training programme enhances individual well‐being in students compared to a nonactive control group, as measured by flourishing and perceived stress.
4. In an exploratory manner, following the theoretical assumption of Stenfors et al. (2025), we investigate whether a change in connectedness is related to individual well‐being.

## METHODS

### Participants

Initially, 204 students participated, comprising 165 women and 38 men; one participant did not disclose their sex. One hundred eleven participants were randomised into the intervention group and 93 into the control group. According to the meta‐analysis by Golden et al. (2021) on mindfulness‐based programmes and self‐compassion in nonclinical populations, the adherence rate was at least 50%, resulting in 80 students participating and attending the intervention four or more times. Nevertheless, the results of the investigation into the influence of mindfulness training on aspects of connectedness and mental health remained unchanged when an intention‐to‐treat analysis was conducted, and 204 participants (111 in the intervention group) were included in the analysis.

In the final sample, 80 participants (13 men, 67 women; mean age: 24.71 years, SD = 4.85) were assigned to the intervention group, and 92 participants (19 men, 73 women; mean age: 24.49 years, SD = 5.19) were assigned to the control group. Most participants were students from the Faculty of Faculties of Human Sciences, Medicine, and Language and Cultural Studies at a University in Bavaria, Germany. Table 1 shows an overview of the characteristics of participants in each group. Ethnicity characteristics were not recorded because only 7.9% of the University were international students.

**TABLE 1:** Descriptive statistics by group (intervention vs. control).

	Intervention (*N* = 80)	Control (*N* = 93)
Age, M (SD)	24.7 (4.85)	24.5 (5.19)
Gender, *n* (%)		
Male	13 (16.2%)	19 (20.4%)
Female	67 (83.8%)	73 (78.5%)
Unknown	–	1 (1.08%)
Physical activity (minutes per week), M		
Pretest	549	727
Posttest	536	645
Field of study, *n*		
Biology and preclinical medicine	5	7
Catholic theology	2	4
Chemistry and pharmacy	3	4
Computer and data science	1	1
Economics	3	4
Humanities	25	22
Language, literature and cultural studies	14	16
Law	4	4
Mathematics	2	–
Medicine	13	11
Philosophy, art, history and social sciences	6	16
Physics	1	4
Unknown	1	–

*Note*: *n* (%) indicates the frequency (percentage) of participants in each group. An endash (−) denotes that no cases were observed in that cell.

Abbreviations: M, mean; *n*, number of participants in each group; *N*, sample size; SD, standard deviation.

The study has been reviewed and approved by the Ethic Research Board of the University of Regensburg. Informed consent was appropriately obtained before completing the questionnaires.

### Material

The internal consistency (Cronbach's Alpha and McDonald's Omega) for all scales in the pre‐ and posttests was good (>.8); see Data S1). The no‐answer rate for the single items was very low, with only 0.3% of all items. They were declared as missing values for the calculation of the mean.

#### Prosocialness scale for adults

(PS, Caprara et al., 2005): Prosocial behaviour was measured with 16 items, which are answered on a 5‐point Likert scale ranging from 1, *Never/rarely true* to 5, *Almost always/always true* (example item: ‘I try to console those who are sad’.). The questionnaire is based on item response theory (IRT). The reliability (*α* = .91), difficulty parameter and discrimination parameter were suitable, and the IRT analyses support the effectiveness and sensitivity of the test (Caprara et al., 2005). For the German version, the questionnaire was forward‐ and backward‐translated.

#### Connectedness to nature scale

(CNS; Pasca et al., 2017): Connectedness to nature was measured with 13 items of the CNS answered on a 5‐point Likert scale ranging from 1, *Strongly disagree* to 5, *Strongly agree* (example item: ‘Like a tree can be part of a forest, I feel embedded within the broader natural world’.). The mean was calculated.

#### Self‐compassion scale

(SCS; Neff, 2003): The short form of the Self‐Compassion Scale was used to measure self‐compassion. The questionnaire assesses how one typically copes with oneself in difficult times. It consists of 12 items, which are assigned to six subscales (self‐kindness, self‐judgement, common humanity, isolation, mindfulness and over‐identification) and is answered on a 5‐point Likert scale ranging from 1, *Almost never* to 5, *Almost always* (example item: ‘I try to be understanding and patient towards those aspects of my personality, I do not like’.)

#### Freiburg mindfulness inventory

(FMI; Walach et al., 2006): Mindfulness was measured using the short version of the Freiburg mindfulness inventory, which has 14 items (answered on a 4‐point scale ranging from almost never to almost ever), including the item ‘I am open to the experience of the moment’.

#### Flourishing scale

(FS; Diener et al., 2010; German version: Esch et al., 2013): The Flourishing Scale consists of eight items (example item: ‘I lead a purposeful and meaningful life’). Participants rate each item on a 7‐point Likert scale, ranging from 1 (*Strongly disagree*) to 7 (*Strongly agree*). The mean of the items was calculated. Esch et al. (2013) supported the reliability and validity of the German scale, with Cronbach's alphas ranging between 0.79 and 0.85.

#### Perceived stress scale

(PSS‐10, Schneider et al., 2020): The German 10‐item Perceived Stress Scale focuses on the thoughts and feelings over the past month. The questionnaire consists of 10 items, which are assigned to two subscales (helplessness and self‐efficacy) and are answered on a 5‐point Likert scale ranging from 1, *Never* to 5, *Very often* (example item: ‘How often have you felt annoyed in the last month about things you could not control?’)

### Procedure

The study design is a randomised controlled trial with a pre‐posttest design, as part of a larger study examining the impact of mindfulness training on job satisfaction parameters. That data are intended to be published somewhere else. An online mindfulness course was advertised to university students through mailing lists, social media sites and posters, and participants were asked to apply by completing a survey. They were paid €10 for participating in the posttest or received course credit. The rerandomisation method was used (Morgan & Rubin, 2012): Eligible study participants were randomly divided into two equal groups, and Fisher's Exact Test was applied to test the null hypothesis of independence from group assignment for each variable. Randomisation was repeated until *p*‐value thresholds of *p* > .05 for perceived stress, flourishing, mindfulness, prosociality, connectedness to nature and self‐compassion, and *p* > .1 for all other variables were met. The final group allocation used the R random seed 8847. At the end of the course, participants were asked to complete the survey again. The questionnaires were given in the order shown in the material part, with the demographic questionnaire being the first, followed by PS, CNS, SCS, FMI, FS and PSS‐10. The control group was a passive group, who did not receive training.

#### Description of the mindfulness course

Each session lasted 90 min, and the course was delivered over 8 weeks, with one session per week. All sessions were conducted live. There were no pre‐recorded components. This format allowed instructors to interact directly with participants and tailor the sessions as needed. While the sessions were delivered separately to each subgroup, the content, themes and structure were identical across all groups.

The main topics of the mindfulness course, which was not strictly a MBSR course, were mindfulness, compassion and connectedness to nature (see Table 2). The elements of mindfulness (Sessions 1–4) were derived from the Mindfulness‐Based Stress Reduction (MBSR) course and the aspects of compassion (Sessions 5–6) from the Mindfulness Self‐Compassion (MSC) course. The elements of the seventh session were derived from the neuroplasticity training by Rick Hanson, and the themes of the eighth session were taken from the MBSR course (‘mountain meditation‘), the MSC course and some ideas from the Thich Nhat Hanh tradition. The last four sessions were chosen because the internal transformative qualities, especially connectedness, are specifically targeted for training through this course. Two experienced teachers of MBSR and Mindfulness‐Based Self‐Compassion taught the courses. Each session began with a guided mindfulness practice, followed by an exchange in dyads or small groups. This was complemented by an inquiry process within the larger group, which included shared reflections and discussions of questions. A brief theoretical introduction was provided to introduce the weekly topic, and a second brief practice session was offered before the session closed. Weekly assignments supported the development of a regular personal practice and typically included uploading 15–20‐min meditation recordings. The mindfulness course was conducted online via live Zoom sessions. The intervention group was divided into five subgroups to maintain manageable class sizes. One group met on Mondays, while two groups met on Tuesdays and two groups met on Thursdays. Each participant was assigned to one of these subgroups and only interacted with their assigned group. Group sizes ranged from 29 to 31 people.

**TABLE 2:** Overview of the mindfulness course.

Week	Topic	Content & Practices	Homework/uploads	Key concepts/learning objectives
1	What is mindfulness?	**Practice:** Raisin exercise (reflecting on the journey from seed to consumption); body scan (body as the first foundation of mindfulness) **exchange/inquiry:** Getting to know each other; group formation; discussion in dyads or small groups **input:** What is mindfulness?	Body scan, mindful eating, solving the 9‐dot puzzle **upload**: Body scan, 9‐dot puzzle	Foundations of mindfulness Body awareness Mindful consumption Reflecting on interconnectedness (food journey)
2	Thoughts/perception	**Practice:** Body scan; breath meditation; sitting postures; observing thoughts **exchange/inquiry:** Sharing and reflection in dyads or groups **input:** Thoughts and perception	**Practice:** Body scan, breath, body awareness; explore unpleasant events **upload:** Breath meditation, table of pleasant events	Observing thoughts nonjudgmentally Awareness of perception Linking experience and reflection
3	Feelings/stress response	**Practice:** Breath, body, sounds; STOP exercise; vagus nerve breathing exercise **exchange/inquiry:** Sharing stressful experiences; group reflection on stress signs **input:** Emotions and stress; mindfulness triangle	**Practice:** Body, breath, sounds; reflect on stressors and handling patterns; mindfulness in daily life **upload:** Meditation on breath, body and sounds	Emotional and stress awareness Mindfulness tools for stress regulation Body‐based coping mechanisms
4	Continuing on stress responses: Resistance	**Practice:** Mindfulness in movement (yoga); walking meditation; breath observation; STOP exercise **exchange/inquiry:** Group sharing and reflection on resistance and suffering **input:** Concept of resistance; suffering = pain × resistance; creating space between stimulus and reaction; introduction to observing thoughts and feeling emotions	**Practice:** Movement (yoga, walking), breath, thoughts, emotions; STOP exercise; explore the space between stimulus and reaction; mindfulness in daily life	Understanding the role of resistance in suffering Cultivating mindful awareness of emotions; recognise and cultivate mindful space between stimulus and reaction to reduce reactivity
5	Self‐compassion: Activation through touch and speech	**Practice:** Body, breath, thoughts, emotions (noticing resistance); hand gestures; affirmations; loving‐kindness meditation for a friend and oneself **exchange/inquiry:** Dyadic/group sharing on self‐care experiences **input:** Self‐compassion; shared humanity	**Practice:** Hand gestures, loving‐kindness meditation, self‐care in daily life **upload:** Loving‐kindness meditation	Cultivating self‐compassion through physical and verbal cues Recognising shared humanity as basis for empathy and connection
6	Self‐compassion: Activation through images	**Practice:** Loving‐kindness meditation (friend, self, neutral person); guided imagery: Safe place with a helper figure **exchange/inquiry:** Sharing with mindful listening in dyads or groups **input:** Continued development of self‐compassion through imagery	**Practice:** Loving‐kindness, safe place visualisation, hand gestures, caring activities; metta for neutral persons; exploration of pleasant events **upload:** Safe place visualisation, table of pleasant events	Deepening self‐compassion through guided imagery Expanding compassion beyond self Use of guided imagery to enhance self‐compassion and internal safety through symbolic representation
7	Positive neuroplasticity: Cultivating joy and gratitude	**Practice:** Loving‐kindness in movement; loving‐kindness meditation (for a friend, oneself, a neutral person and all beings); joy and gratitude practices **exchange/inquiry:** Sharing pleasant experiences and reflections **input:** Joy, gratitude, sympathetic joy as counterbalance to negativity; concept of positive neuroplasticity	**Practice:** Focus on moments of joy, journaling about gratitude and joy; random acts of kindness	Enhancing emotional resilience through joy, gratitude and sympathetic joy Understanding and applying positive neuroplasticity Cultivating prosocial emotion
8	Integration/connectedness with nature and all beings/self‐compassion as the basis for universal compassion	**Practice:** Mindfulness in movement; mountain meditation; sensory anchor exercise (5–4–3‐2‐1: Seeing, hearing, feeling, smelling, tasting); loving‐kindness meditation for oneself, others and the planet **exchange/inquiry:** Group reflection: ‘What do I take with me? What do I want to remember?’ **input:** Recognising shared humanity, interdependence and connectedness with nature **integration/closing:** Reflection on how to carry forward the experience	**Upload:** Mountain meditation	Integration of mindfulness and compassion into daily life Deepening sense of interconnection with all beings and nature Self‐compassion as foundation for universal compassion

Assuming a small to moderate effect of *f* = 0.15, an alpha level of *α* = .05, a power of 1‐*ß* = .80, two groups (mindfulness group and control group), and two measurement points, *n* = 90 students should have to be recruited. Two hundred four students could be recruited initially for both groups. However, as mentioned earlier, only 80 participants attended four times or more. The study was preregistered on the Open Science Framework (https://osf.io/rj3sg/overview?view_only=830afe7b3c6a4e9383b86b153bc67412).

### Statistical analysis

SPSS 29 was used for the statistical analysis. A repeated‐measure analysis with the within‐subject factor time (pre‐ and posttest) and the between‐subject factor group (intervention, control group) was conducted for each dependent variable. To analyse the factors of connectedness (prosocialness, connectedness to nature and self‐compassion), alpha error was corrected (Bonferroni method) and set to .0167. Regarding the parameters of individual well‐being (flourishing and stress reduction), the correction results in an alpha error of .025. *T*‐tests were applied to the post hoc comparisons. Homogeneity of variances was asserted using Levene's test, which showed that equal variances could be assumed for all pre‐ and posttests for all variables. Almost all pre‐ and posttests of the variables were normally distributed, as assessed by the Shapiro–Wilk test, except for the pretests of mindfulness and self‐compassion, and the posttests of flourishing and perceived stress.

For the exploratory analysis, if a change in the inner transformational qualities is related to the individual well‐being, two regressions (Enter method) were conducted with the criteria of flourishing and perceived stress (change score between the pre‐ and posttest) and the predictors group, the difference scores of the pre‐ and posttest of self‐compassion, prosociality, connectedness to nature and mindfulness. As demographic variables, gender and age were included. This exploratory analysis was not preregistered. The exact tables of the regression analyses were presented in Table S2. In addition, two mediation analyses using the PROCESS macro of Hayes (2018), which uses ordinary least square regression, were performed to investigate direct and indirect effects.

## RESULTS

Table 3 presents the means and standard deviations for each group and time across the six dependent variables.

**TABLE 3:** Means and standard deviations for key measures by group (intervention vs. control) and time of evaluation (pretest vs. posttest).

	Intervention M (SD)		Control M (SD)	
	Pretest	Posttest	Pretest	Posttest
Connectedness to nature	3.25 (0.59)	3.50 (0.60)	3.38 (0.60)	3.45 (0.70)
Prosociality	4.03 (0.44)	4.07 (0.48)	3.95 (0.48)	4.02 (0.47)
Self‐compassion	2.75 (0.69)	3.13 (0.59)	2.74 (0.58)	2.86 (0.61)
Mindfulness	2.49 (0.45)	2.77 (0.36)	2.45 (0.42)	2.58 (0.46)
Flourishing	5.29 (0.81)	5.49 (0.75)	5.39 (0.71)	5.52 (0.82)
Stress	3.19 (0.66)	2.97 (0.54)	3.15 (0.63)	3.09 (0.66)

Abbreviations: M, mean; SD, standard deviation.

**FIGURE 2:**
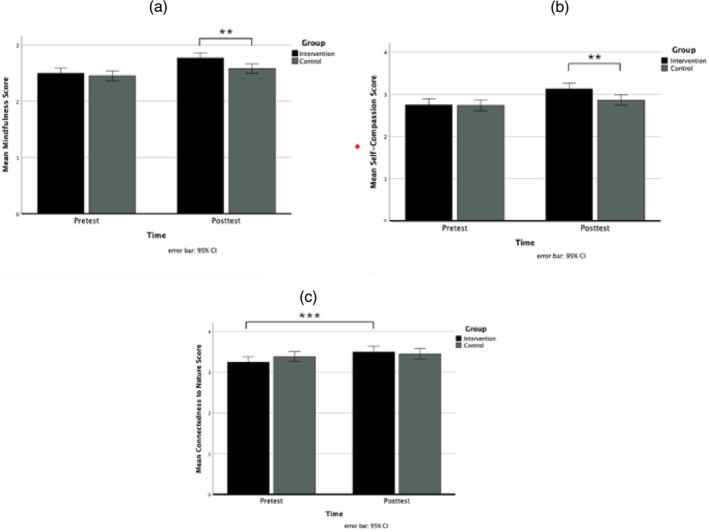
(a,b,c) mean mindfulness score (2a), mean self‐compassion score (2b) and mean connectedness to nature score (2c) dependent on time and group.

For the dependent variables, mindfulness, self‐compassion and connectedness to nature, a significant interaction effect was found for the factors time and group (see Figure 2a–c). Figure 2a shows that, regarding mindfulness, the groups did not differ in the pretest (*p* = .495) but did in the posttest (*p* = .003), with higher values for the intervention group. The same holds for self‐compassion (Figure 2b): The groups did not differ in the pretest (*p* = .889) but did in the posttest (*p* = .004), with higher values for the intervention group. The interaction effect for connectedness to nature was that the CNS scores between the pre‐ and posttests differed only for the intervention group (*p* < .001), but not for the control group (*p* = .137), as seen in Figure 2c.

All other interaction effects were not significant.

Table 4 shows the ANOVA results for the dependent variable.

**TABLE 4:** Repeated‐measures ANOVA for differences between the two groups (mindfulness and control group [CG]) in the different dependent measurements.

Dependent variable	Main and interaction effects	*F* (*df*_eff_,*df*_err_)	*p*	*η*_*p*_^2^
Mindfulness	Effect of time	40.173 (1170)	<.001	.191
	Effect of group	4.254 (1170)	.024	.048
	Time x group	5.116 (1170)	.025	.029
Prosociality	Effect of time	3.454 (1171)	.065	.020
	Effect of group	.811 (1171)	.369	.005
	Time x group	.185 (1171)	.668	.001
Self‐compassion	Effect of time	43.984 (1171)	<.001	.204
	Effect of group	1.668 (1171)	.107	.015
	Time x group	11.019 (1171)	.001	.061
Connectedness to nature	Effect of time	19.917 (1171)	.001	.104
	Effect of group	.226 (1171)	.635	.001
	Time x group	6.838 (1171)	.01	.038
Flourishing	Effect of time	10.745 (1169)	.001	.060
	Effect of group	.288 (1169)	.592	.002
	Time x group	.475 (1169)	.492	.003
Perceived stress	Effect of time	9.805 (1170)	.002	.055
	Effect of group	.247 (1170)	.620	.001
	Time x group	2.911 (1170)	.090	.017

### The relation between connectedness and individual well‐being

#### The difference in the perceived stress

No evidence of multicollinearity or autocorrelation was found. A multiple regression analysis was conducted with perceived stress as the criterion variable and seven predictors. The overall model was statistically significant and explained 35.7% of the variance in perceived stress, *F* (7, 159) = 3.36, *p* < .001. Among the predictors, the difference scores of self‐compassion (*β* = −.32, *p* < .001) and mindfulness (*β* = −.35, *p* < .001) were the only variables that significantly and negatively predicted perceived stress. The mediation analysis exhibited two significant indirect effects. The indirect effect of group on the difference score on perceived stress through the difference score of self‐compassion (a₁b₁ = 0.0942) was significant, 95% CI [0.0258, 0.1800], as it was in addition the difference score of mindfulness (a₂b₂ = 0.0699), 95% CI [0.0073, 0.1608].

#### The difference in flourishing

No evidence of multicollinearity or autocorrelation was found. A multiple regression analysis was conducted with flourishing as the criterion variable and seven predictors. The overall model was statistically significant and accounted for 33.9% of the variance in flourishing, *F* (7, 159) = 3.47, *p* < .001. Among the predictors, the difference scores of self‐compassion (*β* = .23, *p* = .006) and mindfulness (*β* = .38, *p* < .001) were the only variables that significantly and positively predicted flourishing. The indirect effect of group on the difference score on flourishing through the difference score of self‐compassion (a₁b₁ = −.0733) was significant, 95% CI [−.1492, −.0152], as it was in addition the difference score of mindfulness (a₂b₂ = −.0794), 95% CI [−.1696, −.0077].

## DISCUSSION

The results showed that an online mindfulness programme with elements of mindfulness, self‐compassion and connectedness to nature can enhance these elements compared to a nonactive control group. Nevertheless, there was no effect on stress reduction, flourishing or prosociality. This leads to the conclusion that mindfulness practice can train parts of inner transformative qualities. Furthermore, the enhancement of self‐compassion and mindfulness in this study improves flourishing and reduces perceived stress.

These results align with the theoretical model proposed by Stenfors et al. (2025), which suggests that mindfulness practice can foster transformative qualities, such as connectedness to oneself and nature. This latter part has not been investigated in this study. We did not find an effect of the mindfulness training on connectedness to others, which was operationalised as prosociality. One reason for this might be that the choice of prosociality as one example of connectedness to others is selective. However, it has been demonstrated that trait mindfulness is associated with prosociality (Jansen, Rahe, & Wolff, 2024). Prosociality was also chosen as a concept for connecting to others because it has been demonstrated that mindfulness practice can enhance prosociality (Schindler & Friese, 2022). Variables related to the study design, such as intervention intensity, type of control group or randomisation, did not moderate the intervention effects (Donald et al., 2019). One reason for the lack of effect of the mindfulness intervention might be that the mindfulness course's central themes included elements of the MBSR course and aspects of self‐compassion and connectedness to nature.

Regarding self‐compassion, the results also align with a meta‐analysis showing that mindfulness practice can enhance self‐compassion in a nonclinical sample, with a medium effect size found (Golden et al., 2021). These findings are in line with the study's results, which demonstrated a medium effect size for the impact of mindfulness training on self‐compassion.

With regard to the aspect of connectedness to nature, the results align with another study that has shown, among other things, that a 7‐day app‐based compassion meditation training led to more substantial changes in nature connectedness in the meditation group (Loy et al., 2024). Also, the programme in this course included elements of (self)compassion. Even a brief mindfulness or loving‐kindness meditation could improve nature connectedness compared to an active control group that received a progressive muscle relaxation session (Aspy & Proeve, 2017). Because connectedness to nature was an integral element of the mindfulness programme (see Table 2), the observed effects on connectedness to nature represent a near effect, reflecting transfer to an outcome that was directly targeted by the intervention.

Although the mindfulness practice did not improve stress reduction and flourishing, the improvement in self‐compassion and mindfulness was related to enhanced stress reduction and flourishing, highlighting the relationship between these concepts but not establishing causality. In other words, the study could prove the relationship between transformative qualities and individual well‐being. This aligns with the theoretical model of Stenfors et al. (2025), which posits that mindfulness practice can cultivate transformative qualities, such as self‐connection, and these qualities are related to mental well‐being. The lack of effect of the mindfulness training on the reduction of stress and improvement of flourishing seemed to be astonishing at first glance because, for example, a meta‐analysis of randomised controlled trials with online mindfulness intervention has shown that there was a medium effect for perceived stress at the post‐intervention (Jayawardene et al., 2017). One reason might be that this study's mindfulness programme focused on connectedness rather than stress reduction. The limited impact of mindfulness training on flourishing is evident in an updated meta‐analysis of digital randomised controlled trials (Sommers‐Spijkerman et al., 2021), which reveals only a small effect when outliers and low‐quality studies are excluded. In the study presented here, outliers were not omitted, rerandomisation was used, and the sample size was sufficient. However, as mentioned in the introduction, we conceptualised flourishing as a comprehensive measure of well‐being. It is possible that the mindfulness training had more specific effects on particular aspects of well‐being, such as hedonic well‐being.

### Limitations

Even though this is a well‐powered study with re‐randomisation, there are several limitations: the control group is a nonactive control group. The study focused on students; however, most were from the humanities, medicine and language and cultural studies. As in the study by Loy et al. (2024), most of the students were female, and it seemed worthwhile to investigate why participation is less attractive to male students. Furthermore, connectedness with others has been studied primarily, including the variable of prosociality; but other relevant concepts, such as empathic concern or compassion, may also be appropriate.

### Implications

The results of our study suggest that the conceptual model proposed by Stenfors et al. (2025) requires further development. The influence of meditation practice on transformative qualities can be at least partly confirmed. However, the impact on individual well‐being is not confirmed, though the relationship between transformative qualities and individual well‐being is. From a theoretical point of view, it is essential to examine the impact of mindfulness training on transformative qualities in greater depth, before investigating the influence of mindfulness practice on transformative qualities and well‐being, as well as sustainable behaviour. However, given the time left to combat the climate crisis, it might be more useful to study concrete interventions which effectively promote meaningful individual actions.

### Conclusion and future research

This study hints that mindfulness practice can enhance connectedness to oneself, to others and to nature as one aspect of transformative qualities. The improvement, at least in terms of connectedness to oneself, is related to individual well‐being. Although the practice and effectiveness of internet‐mindfulness interventions are well‐established (Zhang et al., 2020), it would be interesting to replicate the study within a presence mindfulness training framework, as connectedness is a key aspect of teaching in presence. Furthermore, it would be necessary to investigate the influence of moderating variables on the effects of mindfulness training. Moderating variables might be personal trait factors. It has been shown that openness, agreeableness and curiosity predict positive expectancy of meditation, whereas neuroticism predicts higher perceived barriers (Ryakhovskaya et al., 2025). Other moderating factors in the relationship between mindfulness training and connectedness to others may include improved executive control, enhanced self‐regulation abilities and increased empathic concern, as suggested by the systematic review and meta‐analysis by Donald et al. (2019). It remains an open question whether, for example, tailored mindfulness training with elements of sustainable behaviour can enhance sustainable behaviour and consumption. Further well‐powered studies should investigate the effect of connectedness, mediated by factors such as self‐compassion, compassion, empathy and connection to nature, on sustainable attitudes and behaviour.

## CONFLICT OF INTEREST STATEMENT

The authors declare no conflicts of interest.

## ETHICS STATEMENT

The study was approved by the ethical review board of the University of Regensburg, no: 24‐3721‐101.

## Supporting information


**Data S1.** Supporting Information.

**Table S1.** Regression analysis with the criterion: difference between post‐pretest in perceived stress.
**Table S2** Regression analysis with the criterion: difference between post‐pretest in flourishing.

## Data Availability

Data can be retrieved at osf: https://osf.io/rj3sg/files/osfstorage?view_only=.
