# Supplementary material for: The effect of online mindfulness training on connectedness to oneself, to others and to nature in students
Source: Appl Psychol Health Well Being. 2026 Mar 10;18(2):e70137. doi: 10.1111/aphw.70137 (PMC12974555; doi:10.1111/aphw.70137)
Supplement: Supplementary file 1 — Data S1. Supporting Information. [file APHW-18-0-s001.docx]

**Supplementary Material 1:**

| Test | Time of Test | Cronbach’s Alpha | McDonald’s Omega |
| --- | --- | --- | --- |
| Prosocialness Scale for Adults | Pretest | .842 | .837 |
|  | Posttest | .865 | .859 |
| Connectedness to Nature Scale | Pretest | .839 | .848 |
|  | Posttest | .884 | .892 |
| Self-Compassion Scale | Pretest | .849 | .847 |
|  | Posttest | .859 | .853 |
| Freiburger Mindfulness Inventory | Pretest | .830 | .836 |
|  | Posttest | .859 | .858 |
| Flourishing Scale | Pretest | .828 | .829 |
|  | Posttest | .867 | .868 |
| Perceived Stress Scale | Pretest | .877 | .882 |
|  | Posttest | .871 | .871 |

**Internal consistency for each measurement**
