# Supplementary material for: The effect of online mindfulness training on connectedness to oneself, to others and to nature in students
Source: Appl Psychol Health Well Being. 2026 Mar 10;18(2):e70137. doi: 10.1111/aphw.70137 (PMC12974555; doi:10.1111/aphw.70137)
Supplement: Supplementary file 2 — Table S1. Regression analysis with the criterion: difference between post‐pretest in perceived stress. Table S2 Regression analysis with the criterion: difference between post‐pretest in flourishing. [file APHW-18-0-s002.docx]

| **Table S1**  Regression Analysis with the criterion: difference between post-pretest in perceived stress | | | | | | | |
| --- | --- | --- | --- | --- | --- | --- | --- |
|  | *b* | *SE(b)* | *beta* | *t* | *p* | 95 % CI(*b*) | |
|  |  |  |  |  |  | LL | UL |
| (constant) | -0.013 | 0.315 |  | -0.042 | 0.966 | -0.635 | 0.609 |
| Diff_sc | -0.395 | 0.100 | -0.323 | -3.949 | <0.001 | -0.592 | -0.197 |
| Diff_cn | -0.040 | 0.098 | -0.029 | -0.405 | 0.686 | -0.233 | 0.154 |
| diff_pro | 0.210 | 0.114 | 0.122 | 1.836 | 0.068 | -0.016 | 0.435 |
| Group_2 | -0.029 | 0.083 | -0.023 | -0.353 | 0.725 | -0.194 | 0.135 |
| Age | 0.001 | 0.008 | 0.007 | 0.106 | 0.916 | -0.015 | 0.017 |
| Sex | 0.052 | 0.104 | 0.032 | 0.500 | 0.618 | -0.154 | 0.259 |
| diff_m | -0.522 | 0.125 | -0.352 | -4.183 | <0.001 | -0.769 | -0.276 |
| *Note*. LL: lower level, UL: upper level; diff: difference between post-pretest | | | | | | | |

| **Table S2**  Regression analysis with the criterion: difference between post-pretest in flourishing | | | | | | | |
| --- | --- | --- | --- | --- | --- | --- | --- |
|  | *b* | *SE(b)* | *beta* | *t* | *p* | 95 % CI(*b*) | |
|  |  |  |  |  |  | LL | UL |
| (constant) | -0.240 | 0.333 |  | -0.721 | 0.472 | -0.899 | 0.418 |
| Diff_sc | 0.293 | 0.106 | 0.230 | 2.767 | 0.006 | 0.084 | 0.502 |
| Diff_cn | 0.097 | 0.104 | 0.069 | 0.933 | 0.352 | -0.108 | 0.301 |
| diff_pro | 0.076 | 0.121 | 0.042 | 0.629 | 0.530 | -0.163 | 0.315 |
| Group_2 | 0.116 | 0.088 | 0.088 | 1.315 | 0.191 | -0.058 | 0.290 |
| Age | 0.009 | 0.008 | 0.068 | 1.049 | 0.296 | -0.008 | 0.026 |
| Sex | -0.116 | 0.111 | -0.069 | -1.047 | 0.297 | -0.334 | 0.103 |
| diff_m | 0.589 | 0.132 | 0.380 | 4.455 | <0.001 | 0.328 | 0.850 |
| *Note*. LL: lower level, UL: upper level; diff: difference between post-pretest | | | | | | | |
